# Editorial: A year in review: discussions in B cell biology

**DOI:** 10.3389/fimmu.2023.1214494

**Published:** 2023-05-24

**Authors:** Harry W. Schroeder

**Affiliations:** Division of Clinical Immunology and Rheumatology, Department of Medicine, University of Alabama at Birmingham Heersink School of Medicine, Birmingham, AL, United States

**Keywords:** autoimmune liver diseases, B1 B cells, chylothorax – central venous occlusive disease, computational B cell biology, germinal center B cells, immunoglobulin structure, marginal zone lymphoma

The biosphere of the B cell landscape is exceptionally large. It encompasses the appearance, development, and fate of B cells as they differentiate from progenitor B cells to individual subsets characterized by selective functions and anatomic locations (1). B cells may act directly on the adjacent environment (2) or at a distance through the immunoglobulins they produce. The process of immunoglobulin generation alters germinal DNA sequence, first by cutting and splicing VDJ gene segments into variable domains and then by engaging in a process of hyper somatic mutation that can drastically alter the biologic properties of the affected immunoglobulins (3). Dysregulation of B cell function and tolerance can lead to a wide array of diseases, including autoimmune (4) and lymphoproliferative (5) disorders. This Research Topic aims to spark discussion of issues that fall outside of classic approaches to B cell Biology and thus offer the opportunity to broaden our perspectives.

Wang et al. report the first case of monoclonal IgM elevated nodal marginal zone lymphoma (NMZL) that was complicated by atypical nontraumatic chylothorax. This is an example of a non-immune complication resulting from the anatomic location of a B cell malignancy. Marginal zone lymphoma (MZL) is a group of B-cell lymphomas that originate from the marginal zone of the lymph node and can occur in the spleen, lymph nodes, and mucosal lymphoid tissue, accounting for approximately 10% of all non-Hodgkin’s lymphomas (NHL). The thoracic duct is the largest lymphatic vessel in the human body. Approximately three-quarters of the lymph, or chyle, from the entire body passes through the thoracic duct and enters the systemic (venous) blood supply at the junction of the left subclavian and left internal jugular veins. A chylothorax is the accumulation of lymph in the pleural space. The patient presented with generalized lymphadenopathy with particular enlargement of right hilar mediastinal nodes. The thoracic duct crosses the mediastinum from right to left at the level of the fifth thoracic vertebra. Obstruction, as in this case, of the thoracic duct above this level often results in a left-sided chylothorax. Anti-B cell treatment led to resolution of the lymphadenopathy, and with it the chylothorax.

Amendt and Tybulewicz report on potential interactions between anti-depressants, such as mirtazapine, and B cells dwelling in the liver afflicted with autoimmune liver disease (AILD). Indwelling B cells can harm the liver by producing autoantibodies, presenting self-antigens to T cells, or releasing proinflammatory cytokines. In liver both the anatomic location and the function of the indwelling B cells can lead to organ dysfunction. Of particular note is the role played by indwelling B1 B cells in ameliorating the effect of disease-promoting B 2 cells. B1 B cells produce natural antibodies that can bind and neutralize harmful self-molecules such as oxidized lipids (e.g., oxLDL) or proinflammatory cytokines such as TNFα or IL1-β. Mirtazapine can ‘wash out’ more migratory B2 B cells whilst retaining more slowly migrating B1 B cells by increasing hepatic sinusoidal blood flow. Enrichment of B1 B cells appears to limit liver inflammation and prevent liver injury by either increasing production of hepatic natural IgM with its anti-inflammatory actions or changing patterns of cytokine production directly, or both. This is a classic example of drug repurposing, where a medication originally developed to treat disease in one organ system is serendipitously found to influence a totally different disease process in a second organ system, opening up a new armamentarium for the treatment of disease.

Comparisons of immunoglobulin variable domain sequences first revealed that the primary structure of each V domain contains four regions of relative sequence stability, termed framework regions (FR), and three regions of high sequence variability, termed complementarity determining regions (CDR) (3). Subsequent crystallographic studies revealed that the CDRs are located at the tip of the variable domains and juxtaposed to create the antigen binding site, as classically defined. The FRs thus serve as the scaffolding on which this antigen binding site rests. A key purpose of the scaffolding is to establish the relationship between the heavy chain and light chain V domains (V_H_ and V_L_). Rhodes et al. show that mutations in the FRs can influence the angle of the V_H_ - V_L_ interface and thus influence the flexibility of the antigen binding site, enabling it to sample a wider array of epitope surfaces and structures than would be available to a static V_H_ - V_L_ interface. These findings have implications for the design of therapeutic monoclonal antibodies, which in some cases could benefit but in other cases could have their clinical utility degraded by variation in the antigen binding site surface. This work serves as a signpost for future research on monoclonal antibody development.

Finally, the process of affinity maturation by means of somatic hypermutation in the germinal center is exceedingly complex. Garg et al. report the development of a computational model to assess the germinal center reaction. Using this tool, they find that the extent of selection stringency dictates clonal dominance. Limited antigen availability on follicular dendritic cells is shown to expedite the loss of diversity of B cells as germinal centers mature. Their model predicts that substantial numbers of T follicular helper cells are essential to balancing affinity maturation with clonal diversity, as a low number of T follicular helper cells impedes affinity maturation and also contracts the scope for a diverse B cell response.

In this year in review, these four reports illustrate the wide array of topics in health and disease that can be addressed through the study of B cell biology. These papers whet the appetite for what will be discovered in the coming years.

## Author contributions

The author confirms being the sole contributor of this work and has approved it for publication.
